# Fever: Its Pathology and Treatment by Antipyretics

**Published:** 1891-09

**Authors:** 


FEVER: ITS PATHOLOGY AND TREATMENT BY ANTIPYRETICS. Boylston Prize Essay of Harvard University, July, 1890. By ROBERT AMORY HARE, M. D., B. Sc., Clinical Professor of Diseases of Children and Demonstrator of Therapeutics in the University of Pennsylvania; Physician to St. Agnes' Hospital and to the Children's Dispensary of the Children's Hospital, etc. No. 10 in the Physicians' and Students' Ready Reference Series. Philadelphia and London: F. A. Davis. 1891. Price, cloth, $1.25 net.

In this little book will be found a thorough discussion of the value of antipyretics in the treatment of fever. Hare divides them into three classes: first, those which inhibit the production of fever; second, those which decrease the production and increase the dissipation of bodily heat; third, those which increase the radiation of heat so that the loss is greater than the supply. The first class the author believes to be the ideal, but unattainable, as there are no drugs capable of accomplishing it; the second class comprises most of the antipyretic drugs that are properly employed in the treatment of fever, while the third class are unreliable and harmful. Drugs of the second class are antipyrin, antifebrin, carbolic acid, salicylic acid and quinine; and those of the third class are cardiac sedatives like aconite and antimony. We quote from Hare the following, which possesses a special interest:

Antipyrin stands today foremost in the ranks of the antipyretics, with antifebrin next, while thallin and phenacetin follow, with perhaps a preference for the latter. These conclusions are in regard to the reduction of fever. In pain the arrangement should somewhat be changed. Antipyrin still takes the lead, but phenacetine is quite as useful as antifebrin and seems more safe. Thallin possesses hardly such power. In rheumatism, of course, the salicylates act better than the rest of the class of antipyretics, particularly in reference to the pain and the cure of the disease itself, but the others control the fever of rheumatism in a much more effective manner.

Since this book was published, Dr. Hare has been appointed Professor of Therapeutics and Materia Medica, in the Jefferson Medical College, and his opinions become all the more attractive. This work will be read with interest by physicians as well as students, and is an ably prepared *résumé* of the subject.

